# Complex Routes of Nosocomial Vancomycin-Resistant *Enterococcus faecium* Transmission Revealed by Genome Sequencing

**DOI:** 10.1093/cid/ciw872

**Published:** 2017-02-23

**Authors:** Kathy E. Raven, Theodore Gouliouris, Hayley Brodrick, Francesc Coll, Nicholas M. Brown, Rosy Reynolds, Sandra Reuter, M. Estée Török, Julian Parkhill, Sharon J. Peacock

**Affiliations:** 1 Department of Medicine, University of Cambridge,; 2 Public Health England, Clinical Microbiology and Public Health Laboratory, Addenbrooke’s Hospital, and; 3 Cambridge University Hospitals NHS Foundation Trust, Cambridge;; 4 London School of Hygiene and Tropical Medicine;; 5 British Society for Antimicrobial Chemotherapy, Birmingham;; 6 North Bristol NHS Trust, Southmead Hospital, Bristol; and; 7 Wellcome Trust Sanger Institute, Hinxton, Cambridge, United Kingdom

**Keywords:** vancomycin resistant, *Enterococcus faecium*, transmission, infection control, genome sequencing.

## Abstract

**Background.:**

Vancomycin-resistant *Enterococcus faecium* (VREfm) is a leading cause of nosocomial infection. Here, we describe the utility of whole-genome sequencing in defining nosocomial VREfm transmission.

**Methods.:**

A retrospective study at a single hospital in the United Kingdom identified 342 patients with *E. faecium* bloodstream infection over 7 years. Of these, 293 patients had a stored isolate and formed the basis for the study. The first stored isolate from each case was sequenced (200 VREfm [197 *vanA*, 2 *vanB*, and 1 isolate containing both *vanA* and *vanB*], 93 vancomycin-susceptible *E. faecium*) and epidemiological data were collected. Genomes were also available for *E. faecium* associated with bloodstream infections in 15 patients in neighboring hospitals, and 456 patients across the United Kingdom and Ireland.

**Results.:**

The majority of infections in the 293 patients were hospital-acquired (n = 249) or healthcare-associated (n = 42). Phylogenetic analysis showed that 291 of 293 isolates resided in a hospital-associated clade that contained numerous discrete clusters of closely related isolates, indicative of multiple introductions into the hospital followed by clonal expansion associated with transmission. Fine-scale analysis of 6 exemplar phylogenetic clusters containing isolates from 93 patients (32%) identified complex transmission routes that spanned numerous wards and years, extending beyond the detection of conventional infection control. These contained both vancomycin-resistant and -susceptible isolates. We also identified closely related isolates from patients at Cambridge University Hospitals NHS Foundation Trust and regional and national hospitals, suggesting interhospital transmission.

**Conclusions.:**

These findings provide important insights for infection control practice and signpost areas for interventions. We conclude that sequencing represents a powerful tool for the enhanced surveillance and control of nosocomial *E. faecium* transmission and infection.

## Summary

We generated genome sequence data from 293 *Enterococcus faecium* isolates from patients with bacteremia spanning 7 years in one hospital. Cases were connected to numerous transmission routes, the complexity of which placed them beyond detection by conventional infection control methods.

*Enterococcus faecium* is a leading cause of nosocomial infections in critically ill and immunocompromised patients [1]. In 2009–2010, *E. faecium* was among the top 10 most common microorganisms associated with healthcare-associated infections in the United States [1]. Vancomycin resistance in *E. faecium* (VREfm) was first reported in 1988 [2, 3] and has since become globally disseminated. Infections caused by vancomycin-resistant enterococci (VRE) have limited treatment options, and are associated with higher mortality and healthcare costs compared with those caused by vancomycin-susceptible enterococci [4–8]. Eradication of *E. faecium* from healthcare settings is challenging because it is often carried in the gut by hospitalized patients [9, 10] and can persist in the environment [11, 12]. Furthermore, some hospital infection control measures such as active screening programs of high-risk patients to detect and isolate VREfm carriers are not universally practiced.

The investigation of suspected VREfm outbreaks is triggered when 2 or more VREfm-positive individuals are identified in the same time and place. This epidemiological approach may be complemented by bacterial typing, but available methods lack sufficient resolution to distinguish between isolates belonging to the same genetic lineage [13]. Whole-genome sequencing (WGS) provides greater discrimination between isolates for a range of nosocomial pathogens, but the study of *E. faecium* has lagged behind. The first completed *E. faecium* genome was only published in 2012 [14], and subsequent genome-based studies have involved relatively small numbers of isolates [13, 15–17]. A single study to date has addressed the clinical application of *E. faecium* WGS to patient cohorts, which used a core genome multilocus sequence typing scheme and confirmed the utility of WGS for the study of *E. faecium* outbreaks [18]. Here, we report the findings of a large study that establishes a role for genome sequencing in the investigation of *E. faecium* transmission.

## METHODS

### Microbiology and Sequence Data

A retrospective study was conducted at the Cambridge University Hospitals NHS Foundation Trust (CUH), a tertiary referral center in the United Kingdom with 1170 beds and 350000 occupied-bed-days per year. The average rate of hospital-associated *E. faecium* bacteremia at CUH was approximately 12.5 vancomycin-resistant and 7 vancomycin-susceptible bacteremias per 100000 bed-days between January 2006 and December 2012. All patients with *E. faecium* bloodstream infection between November 2006 and December 2012 were identified using the diagnostic microbiology laboratory database (n = 342) and cross-referenced with the bacterial freezer archive to identify cases with at least 1 stored *E. faecium* isolate (n = 293). Their first stored isolate (200 VREfm, 93 vancomycin-susceptible *E. faecium* [VSEfm]) was sequenced, together with the first stored isolate from 15 patients with *E. faecium* bacteremia at 2 neighboring hospitals (Papworth and Hinchingbrooke) in 2012 (isolates were not available prior to 2012). All 57 CUH isolates from 2012 and an additional 21 CUH isolates from the study have been reported previously [19, 20].

Patient location and clinical data at onset of bacteremia were collected from paper and electronic medical records for all CUH study patients. Additional ward movement data for 1 year prior to the bacteremia were collected for all patients included in the epidemiological investigation of clustered isolates. Bacteremia was categorized as community-acquired, healthcare-associated, or hospital-acquired based on the criteria defined by Friedman et al [21]. Epidemiological data were not available for patients from Papworth and Hinchingbrooke hospitals. The study was approved by the National Research Ethics Service (reference number 12:/EE/0439) and the CUH Research and Development Department.

DNA was extracted using the QIAxtractor instrument (QIAgen, Hilden, Germany). Phenotypic susceptibility to vancomycin was determined using the British Society for Antimicrobial Chemotherapy disk diffusion method [22] or the Vitek2 instrument (bioMérieux, Marcy l’Etoile, France) with the AST-P607 card. DNA library preparation was conducted according to the Illumina protocol [23], and sequencing was performed on an Illumina HiSeq2000 with 100-cycle paired-end runs. Additional WGS data were sourced from the European Nucleotide Archive (ENA) for a further 529 isolates: 73 from a global collection and 456 from the British Society for Antimicrobial Chemotherapy Bacteremia Resistance Surveillance Project (www.bsacsurv.org). Details of all 837 genomes used in this study are provided in Supplementary Table 1.

### Analysis of Sequence Data

Genome assemblies for the 293 CUH isolates and 73 global isolates were combined and were annotated using Prokka, and a pangenome was estimated using Roary [24]. A maximum-likelihood tree based on 69639 single-nucleotide polymorphisms (SNPs) in the 1057 conserved genes (present in 100% of isolates) was created and lineages were assigned to clade A and clade B based on previous descriptions [16]. Subsequent analyses focused on 284 CUH isolates that resided in a clonal expansion of clade A. Sequence reads were mapped to *E. faecium* Aus0004 (ENA accession number CP003351) using SMALT (http://www.sanger.ac.uk/resources/software/smalt/). A “core” genome was created for each isolate (1921355 to 2801515 base pairs [bp], representing 65%–95% of the genome) by removing genes annotated as plasmid-, phage-, IS-, or transposon-related, putative prophages identified using PHAST (phast.wishartlab.com) [25], and recombination identified using Gubbins [26]. Maximum-likelihood trees were created based on SNPs in the core genome using RAxML [27], a midpoint root, and 1000 bootstraps (Supplementary Figures 1 and 2). iTOL [28, 29] and FigTree were used to visualize the trees. Pairwise differences between isolates were calculated based on SNPs in the core genome.

The mutation rate was estimated for a subset of isolates (CUH cluster 1). Sequence reads were mapped to an assembly of the oldest isolate in the cluster using SMALT, and mobile genetic elements and recombination events were identified and removed, leaving a core genome size of 3100085 to 3212187 bp. The presence of a time signal was evaluated using Path-o-gen and the mutation rate estimated using Bayesian Evolutionary Analysis Sampling Trees (BEAST) [30]. The best molecular clock and tree model was selected based on Bayes factors, calculated using a combination of path sampling and stepping stone sampling [31, 32].

The *vanA* and *vanB* genes were detected using in silico polymerase chain reaction (PCR) and published primers [33, 34]. SNPs and gene content of *vanA* transposons were identified by mapping to the reference transposon M97297 from *E. faecium* strain BM4147. DNA from 6 isolates (5 VREfm, 1 VSEfm; Supplementary Table 1) was extracted using the phenol/chloroform method [35] and sequenced using the PacBio RS II instrument. Sequence reads were assembled using HGAP version 3 [36] of the SMRT analysis software version 2.3.0 (https://github.com/PacificBiosciences/SMRT-Analysis), circularized using Circlator version 1.1.3 [37] and polished using the PacBio RS_Resequencing protocol and Quiver version 1 (http://github.com/PacificBiosciences/SMRT-Analysis). Plasmids carrying *vanA* were identified using in silico PCR, as above. Fully assembled plasmids were compared using WebACT (http://www.webact.org) and BLASTn (https://blast.ncbi.nlm.nih.gov). The VSEfm isolate was interrogated for the presence of a *vanA*-negative plasmid, which was compared with the *vanA*-positive plasmid in a closely related VREfm isolate using WebACT and BLASTn.

## RESULTS

### Study Cohort and Bacterial Isolates

A total of 342 patients were identified as having *E. faecium* bacteremia at CUH between November 2006 and December 2012. Of these, at least 1 isolate was available for 293 (86%) cases and were the basis of further analysis. The majority of cases were hospital-acquired (n = 249) or healthcare-associated (n = 42), with only 2 cases fulfilling the criterion for community-acquired infection [21]. The most common specialties at the time of bacteremia were hematology (28%), transplantation (12%), general surgery (11%), hepatology (10%), and pediatric hematology-oncology (10%).

### CUH *E. faecium* Genomes in a Global Context

We defined the phylogeny of the 293 CUH *E. faecium* isolates within the broader genetic context of 73 global *E. faecium* isolates used previously to delineate hospital- and community-associated lineages [16]. A maximum-likelihood tree was constructed based on SNPs in the conserved genes (Supplementary Figure 3). This showed that 291 of 293 (99%) CUH genomes resided within a lineage categorized previously as hospital-associated (clade A) [16], and were interspersed with global isolates. The remaining 2 study genomes (both VSEfm) clustered with those categorized previously as community-associated or commensal (clade B) [16]. Contrary to expectations, both clade B isolates were healthcare-associated, and the 2 community-acquired infections were caused by clade A isolates.

**Figure 1.**
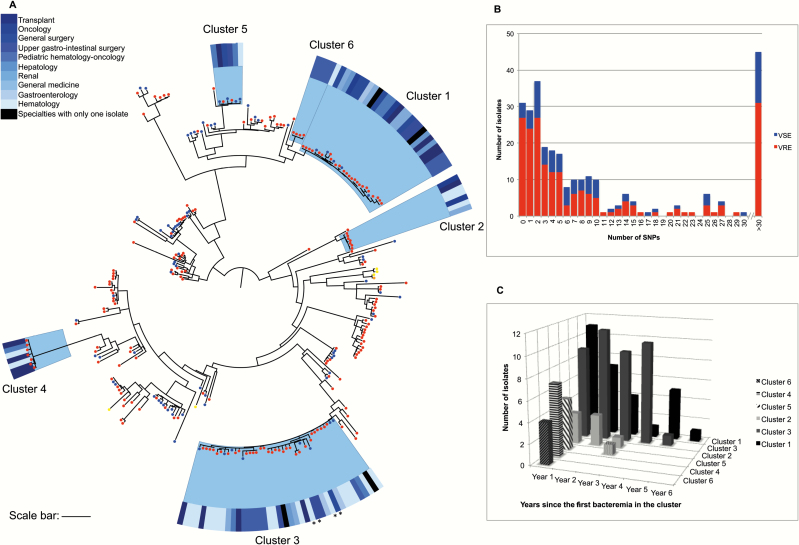
Phylogenetic analyses of *Enterococcus faecium* associated with bloodstream infection in 284 patients at Cambridge University Hospitals NHS Foundation Trust demonstrate numerous independent clades associated with nosocomial transmission. *A*, Maximum likelihood tree of 284 isolates. Colored dots indicate presence of *vanA* (red), *vanB* (yellow), *vanA* and *vanB* (orange), or absence of *van* genes (blue). Clusters 1–6 are used as examples for detailed analyses, and show the medical specialty for each case at the time of bacteremia (blue outer ring). Asterisks indicate isolates in cluster 3 that were not classified as closely related to other isolates in the cluster based on single-nucleotide polymorphism (SNP) differences. Scale bar indicates 84 SNPs. *B*, Pairwise SNP comparison for the 284 isolates shows the closest genetic match for each isolate. Red, vancomycin-resistant *E. faecium* (VREfm); blue, vancomycin-susceptible *E. faecium* (VSEfm). *C*, Number of isolates per year for clusters 1–6 (n = 93), starting from the earliest isolation date (day zero) in each cluster.

We noted that 284 of the 293 CUH genomes formed a highly related clonal expansion within clade A (Supplementary Figure 3). We reconstructed a maximum-likelihood tree for these 284 study isolates based on 4212 SNPs after mapping to a reference and removing recombination (Figure 1A). Overall genetic diversity was high, but the most striking feature was the presence of numerous clusters of highly related isolates. This is indicative of multiple introductions of *E. faecium* into CUH, followed by clonal expansion associated with persistence and transmission over time. Genetic relatedness was also reflected in a systematic pairwise SNP comparison, which showed that 56% of genomes were within 0–6 SNPs of at least 1 other isolate (falling within the estimate for within-host diversity [19]) (Figure 1B). This included 11% that were identical to at least 1 other isolate at the core genome level. Bacteremia represents the “tip of the iceberg” in terms of *E. faecium* burden, and we propose that these were linked to larger covert *E. faecium* populations associated with carriage and/or environmental contamination within this hospital setting.

### Evidence for *E. faecium* Transmission at CUH

We undertook a detailed transmission analysis taking into account patient movement and mutation rate. The *E. faecium* mutation rates reported previously are inconsistent (approximately 5 SNPs/genome/year [15] and approximately 147 SNPs/genome/year [16]). To reconcile this, we estimated the mutation rate for isolates in CUH cluster 1 (Figure 1A) (the second-largest cluster, containing 29 isolates drawn from each year between 2007 and 2012). A BEAST analysis gave an estimated mutation rate of approximately 7 SNPs/genome/year (2.3 × 10^-6^ SNPs/site/year) (Supplementary Figure 4).

We evaluated the relatedness of isolates in 6 exemplar genetic clusters (termed CUH clusters 1–6), which together contained 93 isolates (Figure 1A). The pairwise SNP distance within each cluster after removal of regions of recombination is provided in Supplementary Table 2. The majority of isolates in each cluster were closely related or fell within the estimated mutation rate of 7 SNPs/genome/year, indicating that these patients were epidemiologically linked. The exceptions were 4 of 40 isolates in cluster 3 that were up to 23 SNPs from the closest genetic match. To determine the importance of accounting for recombination in outbreak analyses, we repeated the pairwise comparison of the core genome before recombination blocks were removed (Supplementary Table 2). This demonstrated a predictable increase in median pairwise SNP difference for all 6 clusters by a factor of between 1.9 and 3.6. Taking into account the estimated mutation rate, an additional 19 isolates were >7 SNPs/genome/year apart, demonstrating the importance of accounting for recombination events in these analyses.

Two isolates in cluster 3 had been suspected previously to be part of a ward-based outbreak in 2010 [38], but sequencing revealed that these belonged to a much larger cluster containing isolates from multiple specialties and dating back to 2008 (Figure 1A). This pattern was mirrored in 4 of the 5 other clusters, which contained isolates from numerous specialties (Figure 1A), and 3 clusters that contained isolates drawn from 3 or more years (Figure 1C). These data confirm that transmission had extended far beyond just a single ward and time-point.

**Figure 2.**
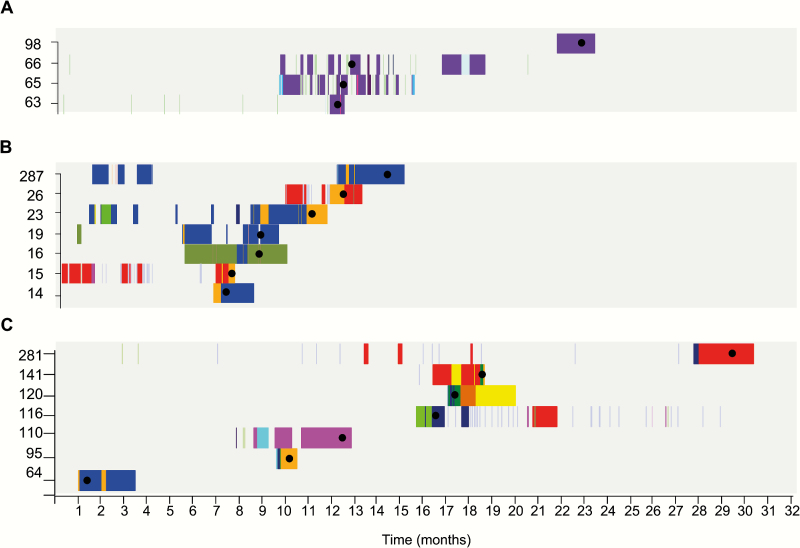
Complex patterns of *Enterococcus faecium* transmission. The combination of genetic *E. faecium* clusters and patient location and movement data over time revealed several patterns of spread. Admission timeline for each patient is shown, with patient ID on the y-axis. Blocks indicate duration of hospital stay. The color of each block is unique to a specific ward or unit. Black dots denote a bloodstream infection, the isolate from which was sequenced. *A*, Patients in cluster 6 involved in a single ward transmission. The first 3 cases on ward A (purple) had overlapping admission dates indicative of an outbreak due to direct or indirect spread. The fourth case occurred many months later. *B*, Isolates in cluster 4 involved in transmission within and between 2 different wards (blue and orange). *C*, Isolates in cluster 2 from numerous wards.

To provide further evidence for the utility of genome sequencing in transmission investigations, we linked genomes with patient ward-movement data for the 6 CUH clusters to visualize the different transmission dynamics. Clusters 2, 4, and 6 are shown in Figure 2 as representatives of the different transmission routes identified. Cluster 6 involved 4 patients on the same ward (1–7 SNPs different [median, 5]) (Figure 2A). Three patients had overlapping admission dates and the fourth had a later, nonoverlapping admission date. This is suggestive of a ward-based outbreak with indirect spread (either from the environment or from unsampled carriers) to the fourth case. Cluster 4 involved 7 patients (1–14 SNPs different [median, 7]) (Figure 2B) and was more complex, involving 2 wards belonging to different specialties located on different floors of the hospital. This provided evidence for direct/indirect transmission between patients sharing the same ward at the same time (eg, patients 14 and 16), and indirect transmission between patients sharing the same ward at different times (eg, patients 23 and 26). Two patients only developed bloodstream infection after transfer to different wards, which obscured links to the likely original source. Figure 2C shows CUH cluster 2, involving 7 patients (1–7 SNPs different [median, 4]). This cluster was unsupported by any clear epidemiological evidence, suggesting widespread dissemination in the hospital, which could be mediated by carriers or represent a communal source.

### Interhospital *E. faecium* Transmission

**Figure 3.**
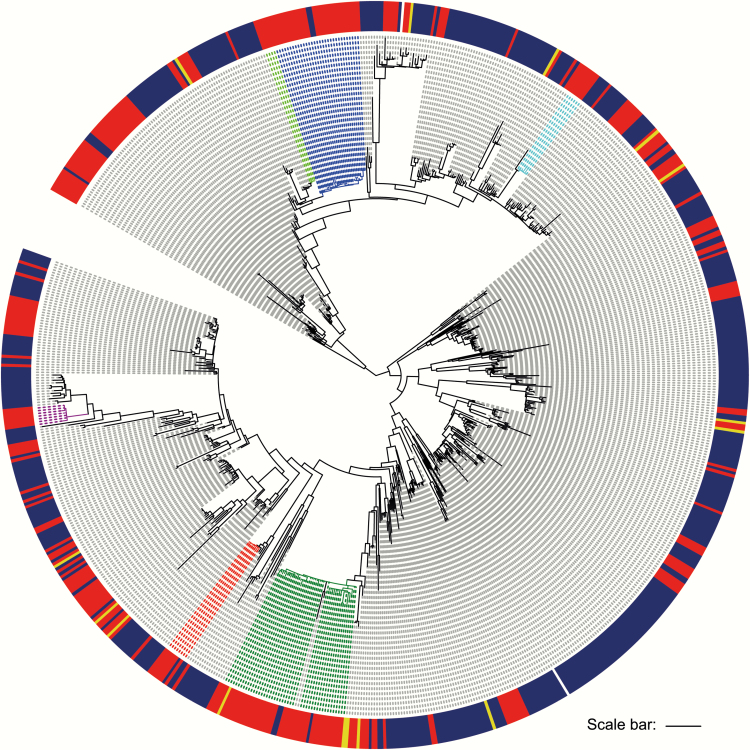
Genetic relatedness between *Enterococcus faecium* from Cambridge University Hospitals NHS Foundation Trust (CUH) patients and a collection from the UK and Ireland. Maximum likelihood tree based on single-nucleotide polymorphisms (SNPs) in the core genome of isolates from the clonal expansion of clade A. Outer ring indicates isolates from CUH (red), 15 isolates from patients with bacteremia in 2 hospitals close to CUH (yellow), and 456 isolates from patients with bacteremia in the UK and Ireland (dark blue). Clusters 1–6 are also indicated (clockwise from 1 o'clock: cluster 5, light blue; cluster 3, dark green; cluster 2, red; cluster 4, purple; cluster 6, light green; cluster 1, dark blue). Scale bar indicates approximately 100 SNPs.

To evaluate the relatedness of *E. faecium* between hospitals at regional and national levels, we combined the genomes from CUH patients with those of 456 *E. faecium* bacteremia isolates from 39 hospitals across the United Kingdom and Ireland between 2001 and 2011, and 15 isolates associated with bacteremia (in 15 patients) at 2 geographically related hospitals, all of which resided in the clonal expansion of clade A. A maximum-likelihood tree demonstrated that CUH clusters 1, 2, and 3 contained isolates from patients at other hospitals, including one from nearby Hinchingbrooke hospital and 6 from other hospitals within our geographic region (East Anglia), indicating regional interhospital transmission (Figure 3). We also identified an isolate that resided in CUH cluster 3 that was originally cultured in the northeast of England, suggesting *E. faecium* transmission between CUH and geographically distant hospitals. CUH isolates were distributed throughout the national collection, supporting the suggestion of multiple introductions into CUH.

### VSEfm and VREfm Are Interspersed in Transmission Clusters

We compared the genomes of the 200 CUH VREfm (197 *vanA* positive, 2 *vanB* positive, and 1 isolate containing both genes) and 93 CUH VSEfm (Figure 1A). Focusing again on clusters 1–6, we found that 3 clusters (1, 3, and 5) contained both VSEfm and VREfm, whereas the remainder contained VREfm alone. To explore the extent to which the gain and loss of resistance was a dynamic process, we compared the genetic relatedness of transposons that carry the gene encoding vancomycin resistance (*vanA*). Four of the 6 clusters each contained >1 transposon type as defined by deletions and/or SNPs (Supplementary Figure 5), suggesting de novo acquisition of vancomycin resistance by hospital-circulating VSEfm lineages. The phylogeny additionally indicated that within each of the CUH clusters 1, 3, and 5, isolates had either gained or lost the transposon on >1 occasion.

To further investigate the loss/gain of *vanA*, we performed long-read sequencing of 6 isolates: 2 from cluster 6 with different transposons based on analysis of short-read data; and 4 from cluster 1, of which 3 had the same transposon based on analysis of short-read data (the earliest [2007], 1 from 2009, and latest [2012] isolate in this cluster), and a VSEfm that was 0 SNPs apart from the 2009 VREfm at the core genome level. Comparison of *vanA*-positive plasmids from cluster 6 confirmed that these were distinct (99% identity but only 51% of the sequence covered [present], Supplementary Figure 6A), indicating the acquisition of >1 *vanA* plasmid by this lineage. In contrast, the 3 *vanA* plasmids from cluster 1 were closely related (99% identity and 100% covered; Supplementary Figure 6B), indicating either a single acquisition event followed by loss by a proportion of the population over time, or multiple acquisition events. Of interest, the plasmid from the earliest isolate from cluster 1 had a 21-kb region that was inverted in comparison to the other 2 plasmids, which included the *vanA* transposon and was located adjacent to a transposase. The VSEfm genome contained a plasmid that was highly related to the *vanA* plasmid carried by the closest genetic VREfm relative (99% identity, 81% covered), with loss of *vanA* being part of an approximate 25-kb deletion. These observations concur with the analysis based on short-read data.

## DISCUSSION

Our study represents an unbiased investigation of *E. faecium* from patients with bloodstream infections over a 7-year period and provides a large-scale overview within a single hospital, and between this and the broader healthcare network. We found that >50% of isolates were highly related to at least 1 other isolate. The use of patient admission and ward movement data allowed us to identify transmission events in individual wards, but combining this with genome data also revealed more complex links between cases that would not have been detected by epidemiological investigation alone. This included patients who appeared to acquire their infecting isolate in one ward but developed bloodstream infection after transfer to another ward, and more complex routes created by patient movement through multiple wards. This implies that targeted infection control interventions triggered by outbreak investigations would only be partially effective.

Our findings are supported by previous studies that reported *E. faecium* with the same pulsed-field gel electrophoresis type on >1 ward [39, 40], suggesting that the complex transmission routes identified in this study exist elsewhere. Additionally, interhospital transmission has been suggested in both Denmark and Australia based on WGS data [13, 15], indicating that this is a globally relevant problem. To date, within-hospital WGS studies have been focused in Australia [15, 17], where the *vanB* type of vancomycin resistance predominates. These studies identified polyclonal hospital populations, supporting our finding of multiple introductions, and hinted at the presence of within-hospital VRE clones, although small isolate numbers (7 and 4 isolates) limited the power to draw robust conclusions from this. Our study extends current knowledge by using more than double the number of isolates used in previous studies to provide the largest and most comprehensive investigation of inter- and intrahospital dynamics of VRE to date, and the first high-resolution insight into the *vanA* setting.

A limitation of our work was its restriction to bacteremia isolates, which constitutes a fraction of the overall VRE burden. Carriers, healthcare workers, and environmental surfaces provide a large “silent” reservoir for transmission [12, 41, 42]. As a result, the prevalence of intra- and interhospital transmissions are likely to be much higher than reported here. Nonetheless, our findings have important implications for hospital infection control. First, the scope of infection control investigations for suspected VREfm outbreaks requires the inclusion of multiple wards and specialties, the potential complexity of which could be simplified by bacterial sequencing. Second, our findings indicate a need to review guidelines to include VSEfm in VREfm control measures since vancomycin resistance appears to be repeatedly introduced into the vancomycin-susceptible population of hospital-adapted lineages, as documented previously for *vanB*-positive VREfm in Australia [15]. Third, our data revealed multiple introductions of *E. faecium* into CUH with subsequent dissemination, emphasizing the importance of active screening to detect carriers in conjunction with isolation to prevent onward transmission. Active screening reduces the rates of VRE infection [43], and countries that implement this strategy (eg, Finland and the Netherlands) have considerably lower rates of VRE infection than the United Kingdom [44].

We conclude that the introduction of routine WGS of *E. faecium* into hospitals would represent a powerful adjunct to existing infection control methods.

## Supplementary Material

Supplementary Data

## Notes

***Acknowledgments.*** We thank Kim Judge; Elizabeth Blane; the Wellcome Trust Sanger Institute library construction, sequence, and core informatics teams; and the staff at the Cambridge Public Health England Microbiology and Public Health Laboratory for their technical input. We also thank Julie Russell (National Collection of Type Cultures) and the British Society for Antimicrobial Chemotherapy for generous provision of isolates.

***Disclaimer.*** The views expressed in this publication are those of the author(s) and not necessarily those of the UK Department of Health or the Wellcome Trust.

***Financial support.*** This work was supported by grants from the Health Innovation Challenge Fund (grant numbers WT098600 and HICF-T5-342), a parallel funding partnership between the UK Department of Health and the Wellcome Trust. This project was also funded by a grant awarded to the Wellcome Trust Sanger Institute (grant number 098051). M. E. T. is a Clinical Scientist Fellow supported by the Academy of Medical Sciences and the Health Foundation.

***Potential conflicts of interest.*** All authors: No potential conflicts.
