# Supplementary material for: Complex Routes of Nosocomial Vancomycin-Resistant Enterococcus faecium Transmission Revealed by Genome Sequencing
Source: Clin Infect Dis. 2017 Feb 23;64(7):886–93. doi: 10.1093/cid/ciw872 (PMC5439346; doi:10.1093/cid/ciw872)
Supplement: Supplementary Data [file ciw872_Supplementary_Data.zip › Raven_Supplementary_Data.docx]

**Supplementary Materials**

**Complex routes of nosocomial vancomycin-resistant *Enterococcus faecium* transmission revealed by genome sequencing**

Kathy E. Raven, Theodore Gouliouris, Hayley Brodrick, Francesc Coll, Nicholas M. Brown, Rosy Reynolds, Sandra Reuter, M. Estée Török, Julian Parkhill, Sharon J. Peacock

**Supplementary Figure 1……………………………………………………………2**

**Supplementary Figure 2……………………………………………………………3**

**Supplementary Figure 3……………………………………………………………4**

**Supplementary Figure 4……………………………………………………………5**

**Supplementary Figure 5……………………………………………………………6**

**Supplementary Figure 6……………………………………………………………7**

**Supplementary Table 2…………………………………………………………..…8**

**Supplementary Figure 1. Bootstrap supports for the phylogeny shown in Figure 1.** Maximum likelihood tree of 284 CUH isolates with 1000 bootstraps. Bootstraps over 90% are shown in blue. Scale bar indicates ~84 SNPs.

**Supplementary Figure 2.** **Bootstrap supports for the phylogeny shown in Figure 3.** Maximum likelihood tree of 284 CUH isolates, 15 isolates from neighboring hospitals to CUH and 456 isolates from across the United Kingdom and Ireland obtained the BSAC collection, with 1000 bootstraps. Bootstraps over 90% are shown in blue. Scale bar indicates ~96 SNPs.

**Supplementary Figure 3. Phylogenetic tree combining genomes of a global collection of *E. faecium* and isolates associated with bacteremia in a single hospital in the United Kingdom.** Maximum likelihood tree based on SNPs in the 1,057 genes conserved across the 293 CUH study isolates and 73 global isolates from a study by Lebreton *et al.* (17). Branch colors indicate Clade A (red) and Clade B (blue). Inner colored ring indicates the clonal expansion of Clade A used in a detailed genetic analysis of transmission networks and *vanA* transposons. Outer colored ring shows whether the CUH study isolates were hospital-acquired (red), healthcare-associated (yellow) or community-acquired (blue). Scale bar indicates ~14,000 SNPs.

**Supplementary Figure 4.** **Estimating the mutation rate of *E. faecium*. A**. Path‐o‐Gen plot of the root-to-tip distance over time for CUH Cluster 1 (n=29). **B**. BEAST tree for CUH Cluster 1 (n=29). Blue bars indicate 95% highest probability density intervals.

**Supplementary Figure 5**. Comparison of *vanA* transposons within *E. faecium* CUH clusters. Left hand side: Maximum likelihood tree based on SNPs in the core genome for CUH isolates in the clonal expansion of Clade A. Top: blue blocks show genes in the *vanA* transposon. Middle: black horizontal bars show the presence (black) and absence (white) of regions of the *vanA* transposons in Clusters 1-6. Right hand side: Vertical bar shows those isolates with SNP(s) in the transposon compared to other transposons in that cluster. Scale bar indicates 62 SNPs.

**Supplementary Figure 6. Comparison of *vanA* positive plasmids based on long-read sequence data. A.** WebACT comparison of the *vanA*-positive plasmid in two isolates from CUH Cluster 6 with matches greater than 100bp shown. **B.** WebACT comparison of the *vanA*-positive plasmid in three isolates from CUH Cluster 1 with matches greater than 999bp shown.

| **Cluster** | **Isolates per cluster** | **Median (range) pairwise SNP difference (recombination removed)** | **Size of genome used in analysis** | **Median (range) pairwise SNP difference ­­­(recombination not removed)** | **Time period (days)** |
| --- | --- | --- | --- | --- | --- |
| 1 | 29 | 10 (0-35) | 2195857 - 2203433 | 21 (0-93) | 1972 |
| 2 | 7 | 4 (1-7) | 2083172 - 2125029 | 9 (2-37) | 854 |
| 3 | 40 | 16 (0-100) | 1973326 - 2148735 | 30 (0-2313) | 1467 |
| 4 | 7 | 7 (1-14) | 2146548 – 2148330 | 13 (3-218) | 212 |
| 5 | 6 | 7 (2-14) | 2011658 – 2025460 | 17 (2-105) | 891 |
| 6 | 4 | 2.5 (1-7) | 2170750 - 2179243 | 9 (3-12) | 324 |

**Supplementary Table 2. Pairwise SNP differences in CUH *E. faecium* Clusters 1-6.** Table showing the number of isolates in each cluster, the median (range) pairwise SNP difference in the core genome after removal of recombination (and size of genome used in the analysis) and prior to removal of recombination, and the time period over which the cluster was isolated.
